# Influence of Modified Starch Admixtures on Selected Physicochemical Properties of Cement Composites

**DOI:** 10.3390/ma15217604

**Published:** 2022-10-29

**Authors:** Marta Sybis, Emilia Konował

**Affiliations:** 1 Department of Construction and Geoengineering, Poznan University of Life Sciences, Piatkowska 94 E, 60-649 Poznan, Poland; 2 Institute of Chemistry and Technical Electrochemistry, Poznan University of Technology, Berdychowo 4, 60-965 Poznan, Poland

**Keywords:** modified starches, natural admixtures, rheology of cement slurries

## Abstract

The conducted research aimed to evaluate the influence of admixtures of various modified starches on the rheological properties of cement composites and their influence on the compressive strength of hardened cement mortars. The study involved 17 different modified starch admixtures. Using a rheometer, the values of viscosity and tangential stresses were determined depending on the shear rate, and were subsequently used to determine the yield point and plastic viscosity of cement slurries. The next parameters tested were the flow of fresh cement slurry and the compressive strength of hardened cement composite. The highest fluidisation was recorded for retentate LU-1420-0.5%Ac-R, an increase of 82%. The conducted tests led to the conclusion that admixing cement composites with modified starches changes the rheological parameters and the compressive strength of cement composites. The highest strength gains occurred for the admixtures of retentate LU-1412-R (increase of 25%). Declines in compressive strength were noticed in the retentate LU-1422-R (decrease by 13%) and the retentate OSA-2.5%-R (decrease of 17%). The admixture of starch hydrolysate significantly decreases the yield point of slurries, which in turn may contribute to the fluidisation of concrete mixes and the reduction of mixing water. The lowest values were obtained for retentates LU-1420-0.5%Ac-R (decrease of 94%), and LU-1412-R (decrease of 93%). It was found that the consistency and compressive strength of cement mortars are affected by both the type of modification and the length of the chemical chain of starch.

## 1. Introduction

The workability of mixes is a major problem in concrete technology. Workability can be understood here as the behaviour of concrete mixes subjected to loads related to implemented technological processes. Designing concrete mixes concerning their workability involves the skilful development of optimal relations between the desired properties of mixtures, the occurring forces, and predicting the behaviour of the systems for the loads applied [1,2,3]. Admixtures, including natural ones, can directly affect the rheological parameters of concrete mixes. 

Currently, environmentally friendly products are growing in importance. Despite the prevalence of inexpensive toxic substances or derivative petrochemical products that have a destructive effect on the planet, “green” products are increasingly chosen all over the world despite the fact that their prices are sometimes higher. These products include biopolymers, biopolymer-stabilised nanoparticles and waste materials [4,5,6,7,8,9,10].

The idea of green or sustainable concrete obtained from plants was common in early times. Numerous ancient structures, which have survived to this day, confirm the influence of natural substances on the durability of buildings constructed with their use [11].

Starch is a material of natural origin, completely biodegradable, and commonly applied in many industries. Most often, it is used as a thickener or stabiliser [12].

Starch is a very important resource that occurs naturally on Earth. To improve its properties, it can be chemically modified (by adding various functional groups to it), physically or enzymatically. The chemical treatment of starch can be divided into several main types of modification: esterification, etherification, oxidation and hydrolysis. Starch esters include sodium starch octenyl succinate, acetylated starches, monostarch phosphates and distarch phosphates. By introducing an alkyl substituent in place of hydrogen in the hydroxyl group, starch ethers are formed, which include hydroxypropyl starches, cationic starches, anionic starches and cross-linked starches [13,14].

The rheological properties of cement mixes depend on many factors, primarily including the degree of saccharification, salt content and pH value [15]. Previous studies also found that the viscosity of maltodextrin solutions depended on the degree of their depolymerisation and decreased with an increase in the glucose equivalent [16,17,18]. In turn, Tur et al. [19] described different experiments in which the apparent viscosity increased with an increase in the glucose equivalent.

The interaction of cement and saccharides has not yet been fully determined. Izaguirre et al. [20] found that the molecular weight of the starch can affect the liquefaction of the cement mix. Higher-molecular-weight polymers have the ability to agglomerate, thereby causing the cement mix to thicken, while lower-molecular-weight polymers tend to adsorb on the surface of the cement grains, causing a dispersing effect, thereby reducing the plastic viscosity of the mix. The influence of polysaccharide structures on cement hydration kinetics has been described in previous studies.

The delay of the hydration phenomenon becomes higher with the increase in polysaccharide concentration. Dextrins with lower molecular weights, compared to starch, foster the formation of more of the soluble fraction and, as a result, delay hydration [21]. Based on the above assumptions, some sugars may be more effective in slowing down hydration. It is commonly assumed that this retardation occurs due to sugars that adsorb on the surface of cement-wetting particles and/or on the surfaces of hydration products. Thus, these products form a barrier to subsequent hydration. Adsorption can take place during chelation, when organic molecules form complexes with metal ions in the cement phase. Sugars exhibit the ability to bind to the cement phase depending on the presence of the -COOH group. The characteristic feature of this group is that oxygen molecules can move close to each other, after which chelation occurs. It has been shown that the pH value of cement can affect hydration. Observations have also shown that the hardening temperature of cement changes the hydration rate of pastes containing sugars [22]. The purpose of this study was to determine the influence of modified starch admixtures on the selected rheological parameters of a liquid mix, as well as on the compressive strength of hardened cement mortar [23,24].

This paper aims to investigate the effect of the addition of modified starches on rheology and the selected physicochemical properties of cementitious pastes and mortars. To determine the possibility of using modified starches as plasticizers, experimental research was carried out. In the case of the slurries, the focus was on rheometric tests. For the mortars, the influence of the modified-starch admixture on the compressive strength was assessed. In the rheological tests of the mortars, measurements of the spread of modified-starch-doped mixtures were performed.

The studies, conducted so far by the authors have shown that the addition of starch derivatives to cement composites can significantly affect their physicochemical parameters. Of particular importance are starch hydrolysates. The addition of dextrin substantially improves the workability of fresh cement. Durability tests carried out on cement blocks showed that the tested admixtures can increase the value of compressive durability by almost 20% without reducing the water–cement ratio [25]. In addition, frost-resistance tests conducted on concretes with dextrin admixtures showed improved concrete durability [26]. Initial studies have also shown that cement composites admixed with dextrin-stabilized colloidal silver have high antimicrobial activity against microorganisms [25,27]. However, the authors showed that the silver nanostructures did not substantially affect the mechanical properties of the cement composites, while the type of starch derivative used was the determining factor in this case. This was the reason why the authors decided to test various starch derivatives to check their effects on the rheology and strength of cementitious composites. The fact that the synthesis of silver nanoparticles with selected starch derivatives is quite simple may contribute to the development of admixtures that, in addition to increasing mechanical sustainability, impart bactericidal properties to the produced biocomposites.

Since the elimination of toxic substances contained in some plasticizers or superplasticizers that can migrate into aquatic and soil ecosystems is of high importance, this research concentrates on the use of fully biodegradable natural plasticizers that contribute to sustainability and ecosystem protection.

## 2. Materials and Methods

### 2.1. Materials

The tests were carried out with the use of CEM I 42.5N Portland cement compliant with the European standard PN-EN 197-1. Native modified starches and starch hydrolysates were used as admixtures:

Starches

- Waxy corn starch (hereafter Waxy corn).
- Potato starch:
  - High-amylopectin starch (hereafter High amylopectin);
  - Oxidized starch (hereinafter LU-1404);
  - Acetylated starch (hereafter LU-1420);
  - Acetylated oxidized starch (hereafter LU-1451-0.5%Ac);
  - Acetylated oxidized starch (hereafter LU-1451-2.5%Ac).

Starch hydrolysates

- Products of enzymatic hydrolysis of starch subjected to membrane filtration (ultrafiltration)—filtrates (F) or retentates (R):
  - Acetylated starch filtrates (hereafter LU-1420-0.5%Ac-F and LU-1420-2.5%Ac-F);
  - Acetylated starch retentates (hereafter LU-1420-0.5%Ac-R and LU-1420-2.5%Ac-R);
  - Distarch phosphate filtrate (hereafter LU-1412-F);
  - Distarch phosphate retentate (hereafter LU-1412-R);
  - Acetylated distarch adipate retentate (hereafter LU-1422-R);
  - Starch sodium octenyl succinate (OSA) retentates (hereafter OSA-0.5%-R, OSA-2.5%-R);
  - Maltodextrins (hereafter Maltodextrin DE 4.9, Maltodextrin DE 8.5).

Selected physicochemical properties of starch derivatives used in this research and methods of their synthesis are presented in [28,29,30,31,32,33,34].

The rheological tests of cement slurries were performed on samples containing 0.5% of the modifier in relation to the amount of cement and for w/c ratios of 0.4 and 0.5.

Starches were digested in make-up water using a multi-station CIMAREC and Poly 15 magnetic stirrer produced by Komet.

The aqueous solution of starch admixtures was added to the cement, after which the entire mixture was stirred for 10 min using a mechanical mixer. The composition of the analysed mixes is presented in Table 1.

Cement slurries were tested mainly for the rheological properties of the produced systems. Measurements were taken for modified starches, including dextrins, which were also verified in terms of how the drying method influenced the rheological behaviour of the resultant slurries. The detailed research methodology is described in the points below.

### 2.2. Methods

#### 2.2.1. Rheometric Tests of Cement Slurries

Viscosity measurements were taken with a Thermo Scientific™ HAAKE™ Viscotester™ 550 viscometer equipped with an MV-DIN rotor.

For each sample, the level of viscosity was measured in three stages: in the first stage, the shear rate increased from 10 to 300 rpm, after which the shear rate was kept constant for 10 s, amounting to 300 rpm, and in the third stage, the shear rate was gradually decreased from 300 rpm to 10 rpm.

Based on the viscosity measurements, the rheological parameters of cement slurries were determined using the Bingham model in the form of:

(1) $$\tau =\tau_{0}+\eta_{pl}\cdot \dot{\gamma }$$

where: τ—shear stress of fluid by load (Pa), $\tau_{0}$—yield point of fluid (Pa), $\eta_{pl}$—dynamic viscosity of fluid (Pa·s), $\dot{\gamma }$ = *dy*/*dt*—shear modulus rate of fluid, (1/s) and Herschel–Bulkley model in the form of

(2) $$\tau =\tau_{0}+{\left(\eta_{pl}\cdot \dot{\gamma }\right)}^{\frac{1}{n}}$$

where: *n*—dimensionless rheological parameter while the remaining parameters are the same as in the Bingham model. To obtain the required rheological parameters, an approach based on linear regression was applied [3].

#### 2.2.2. Production of Cement Mortars

In the first step, the mortar preparation procedure consisted in weighing out the required ingredients, i.e., aggregate, cement, water and admixtures in the following amounts: 1350 g, 450 g, 225 g and 2.25 g per portion. In the next step, the measured dose of a selected admixture was dissolved in the mixing water using a magnetic stirrer (in accordance with the PN-EN 196-1: 2016 standard).

Cement mortars were prepared using a mixer with a program module and a device for automatic control of rotation speed around its axis and the axis of the bowl (planetary rotation).

To determine the influence of admixtures on the consistency of cement mortars, the mixes were tested using a flow-table method, in accordance with the PN-EN 196-1: 2016 standard [30].

Testing of the compressive strength of cement mortars was preceded by making beams with dimensions of 0.04 × 0.04 × 0.16 m. Fresh mortar was placed in three-part polystyrene-cement-mortar moulds. After the mortars were placed in the moulds, they were compacted with a shaker and secured against moisture loss.

The beams were deformed 24 h after the batch was made and placed in water at a temperature of 20 ± 1 °C for 28 days. The compressive-strength test was carried out on halves of previously prepared cement beams, in accordance with PN-EN 196-1 [30]. Subsequently, the samples were removed from the water bath, dried with a cloth of excess water and placed in between the compression plates of the DIGICON 2000 strength press by Walter + Bai AG.

## 3. Results

### 3.1. Flow Tests of Cement Mixes

**Figure 1:**
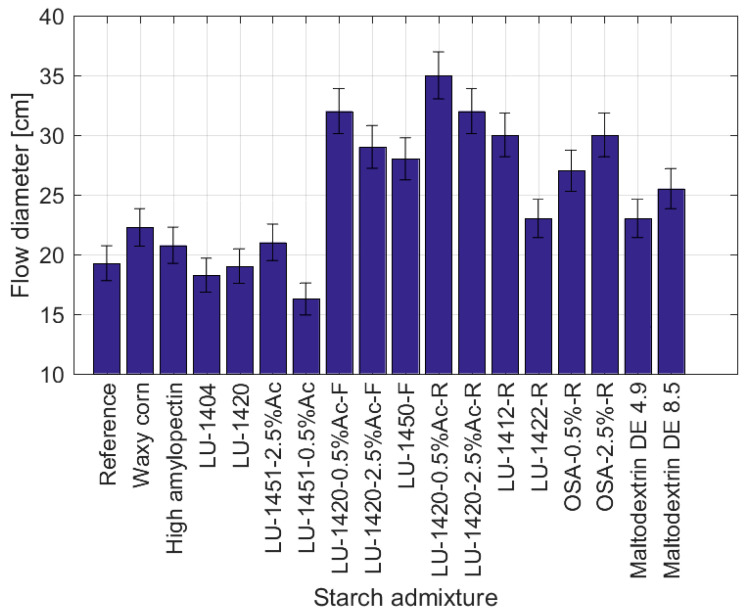
Correlation of the flow diameters of cement mortars with the type of starch admixture used.

Figure 1 shows the results of the flow of fresh cement mortars with the addition of the modified starches. The values were compared to the flow of the reference mortar (without any additives and admixtures). To facilitate the interpretation of the results, the starch hydrolysates were isolated from other admixtures.

The mean reference value of the flow was 19.25 cm. A slight increase in the fluidisation of the mortars was observed for the addition of waxy maize starch (flow 22.5 cm, increase of 17% compared to the reference sample), LU-1451-2.5%Ac (flow 21 cm, increase of 9%) and High amylopectin starch (flow 20.75 cm, increase 8%). A reduction in the fluidisation of the mortars took place with the addition of LU-1451-0.5%Ac (flow 16.25 cm, decrease of 16%) and the oxidized starch LU-1404 (flow 18.25 cm, decrease of 5%). The flow did not change when the acetylated starch LU-1420 was added. With the admixing of the mortars with starch hydrolysates, no reduction in fluidisation was noticed; each admixture improved the workability of the cement mixes. The highest fluidisation was recorded for the retentate LU-1420-0.5%Ac-R (flow 35 cm, increase of 82%), the filtrate LU-1420-0.5%Ac-F (flow 32 cm, increase of 66%), the retentate LU-1420-2.5%Ac-R (flow 32 cm, increase of 66%), the retentate LU-1412-R (flow 30 cm, increase of 56%) and the retentate OSA-2.5%-R (flow 30 cm, increase of 56%). Slightly lower flows occurred for the filtrate LU-1420-2.5%Ac-F (flow 29 cm, increase of 51%), the filtrate LU-1412-F (flow 28 cm, increase of 45%) and the retentate OSA-0.5%-R (flow 27 cm, increase of 40%). Among the samples of hydrolysate, the lowest fluidisation was noted for the Maltodextrin DE 8.5 (flow 25.5 cm, increase of 32%) and for the Maltodextrin DE 4.9 and retentate thickener LU-1422-R (flow 23 cm, increase of 19%).

### 3.2. Rheometric Tests of Slurries Admixed with Modified Starches

**Figure 2:**
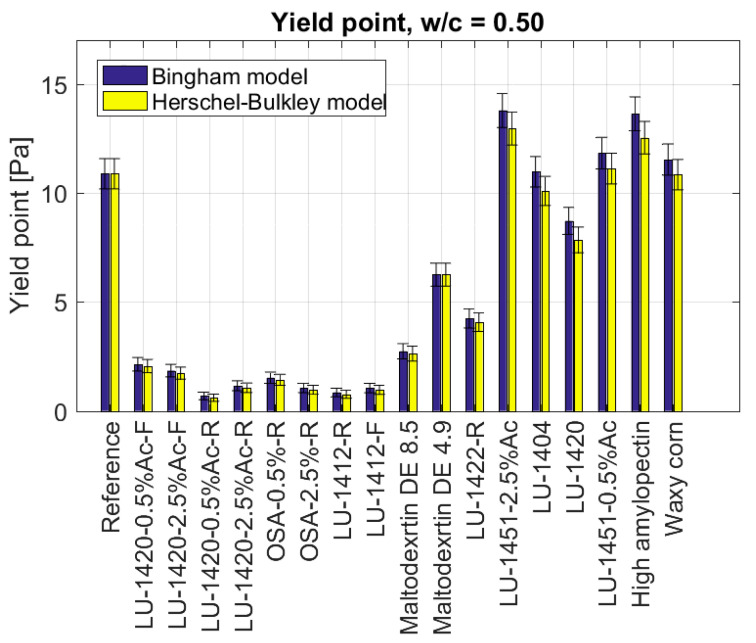
Yield points of cement slurries, depending on the type of admixture in the form of modified starch for w/c = 0.50 starch admixtures used.

The tests with the use of a rotational viscometer were performed for the mortars with 17 different starch admixtures. Using the Bingham and Herschel–Bulkley models, their yield point and plastic viscosity were determined. Figure 2 shows the yield points of the slurries with admixtures of modified starches for w/c = 0.50, excluding the starch hydrolysates. The reference value of the yield point for the slurries without modifiers was *τ*_0_ = 10.9 Pa. An increase was noted in the yield point for two types of modified starch: the starch LU-1450-2.5%Ac (*τ*_0_ = 12.9 Pa, increase of 18%) and the High amylopectin starch (*τ*_0_ = 12.5 Pa, increase of 15%). The value of the yield points, similar to the reference point, occurred for the Waxy corn starch, gelling starch LU-1451-0.5%Ac and oxidized starch LU-1404. Furthermore, for two admixtures of modified starches, a reduction in the yield point was achieved. The LU-1420 reduced the yield point by 28% (*τ*_0_ = 7.8 Pa), whereas the retentate thickener LU-1422-R by 62% (*τ*_0_ = 4.1 Pa) compared to the reference value.

**Figure 3:**
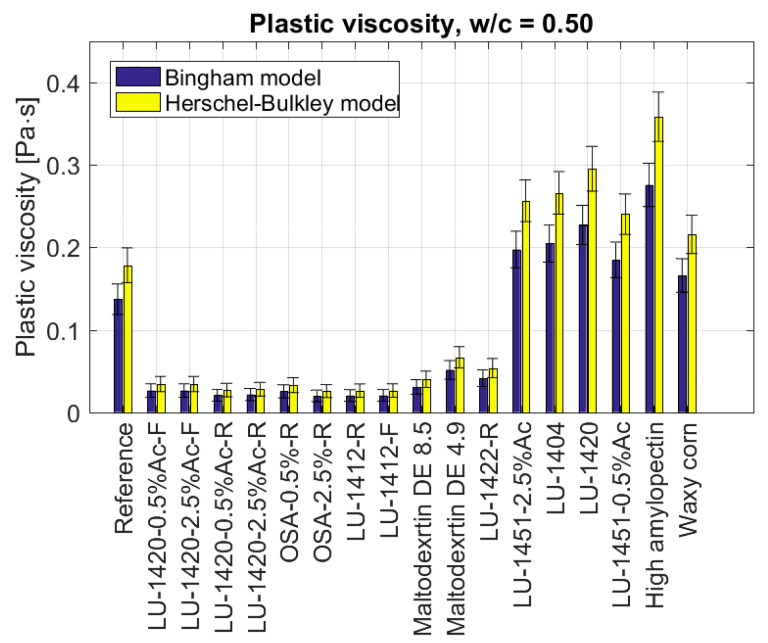
Plastic viscosities of cement slurries depending on the type of admixture in the form of modified starch for w/c = 0.50.

Admixing the mortars with starch hydrolysates lowered the yield point for each of the added starches. The lowest values were obtained with the retentate LU-1420-0.5%Ac-R ($\tau_{0}=0.6\;\mathrm{Pa}$, decrease of 94%), the retentate LU-1412-R ($\tau_{0}=0.8\;\mathrm{Pa}$, decrease of 93%), the retentate OSA-2.5%-R ($\tau_{0}=0.97\;\mathrm{Pa}$, decrease of 91%), the filtrate LU-1412-F ($\tau_{0}=0.97\;\mathrm{Pa}$, decrease of 91%). Slightly higher values for the yield point were recorded with the retentate LU-1420-2.5%Ac-R ($\tau_{0}=1.06\;\mathrm{Pa}$, decrease of 90%), the retentate OSA-2.5%-R ($\tau_{0}=1.4\;\mathrm{Pa}$, decrease of 87%), the filtrate Ac 2.5% $(\tau_{0}=1.7\;\mathrm{Pa}$, decrease of 84%), the filtrate LU-1420-0.5%Ac-F ($\tau_{0}=2.1\;\mathrm{Pa}$, decrease of 81%) and for the Maltodextrin DE 8.5 ($\tau_{0}=2.6\;\mathrm{Pa}$, decrease of 76%). The highest value of the yield point among starch hydrolysates was with the Maltodextrin DE 4.9: $\tau_{0}=6.3\;\mathrm{Pa}$, (decrease of 42%). As in the case of the yield point, the values of the plastic viscosity were similar (Figure 3). For the modified starches, the type LU-1451-2.5%Ac ($\eta_{pl}=0.257\;\mathrm{Pa}\cdot s$, increase of 44%), LU-1420 ($\eta_{pl}=0.295\;\mathrm{Pa}\cdot s$, increase of 66%), High amylopectin starch $\eta_{pl}=0.359\;\mathrm{Pa}\cdot s$, increase of 102%), LU-1404 ($\eta_{pl}=0.266\;\mathrm{Pa}\cdot s$, increase of 49%), gelling starch LU-1451-0.5%Ac ($\eta_{pl}=0.240\;\mathrm{Pa}\cdot s$, increase of 35%) and Waxy corn starch ($\eta_{pl}=0.216\;\mathrm{Pa}\cdot s$, increase of 21%), an increase in plastic viscosity was noted. The reference value for the plastic viscosity was $\eta_{pl}=0.178\;\mathrm{Pa}\cdot s$.

For the starch hydrolysates, a significant reduction in plastic viscosity was observed for all the samples. For the Maltodextrin DE 4.9 and the retentate thickener LU-1422-R, the viscosity values were higher than for the other hydrolysates, amounting to $\eta_{pl}=0.0666\;\mathrm{Pa}\cdot s$ (decrease by 63%). The plastic viscosity of the remaining hydrolysates ranged from $\eta_{pl}=0.0257\;\mathrm{Pa}\cdot s$ (decrease of 86%) for the retentate OSA-2.5%-R to $\eta_{pl}=0.04\;\mathrm{Pa}\cdot s$ (decrease of 78%) for the Maltodextrin DE 4.9.

In the next stage of the research, it was decided to lower the w/c ratio to 0.40. Unfortunately, tests were not successfully performed on all the substances. The slurries admixed with modified starches in combination with the reduced water use could not be thoroughly mixed or, due to their too high density, could not be tested with a rheometer.

**Figure 4:**
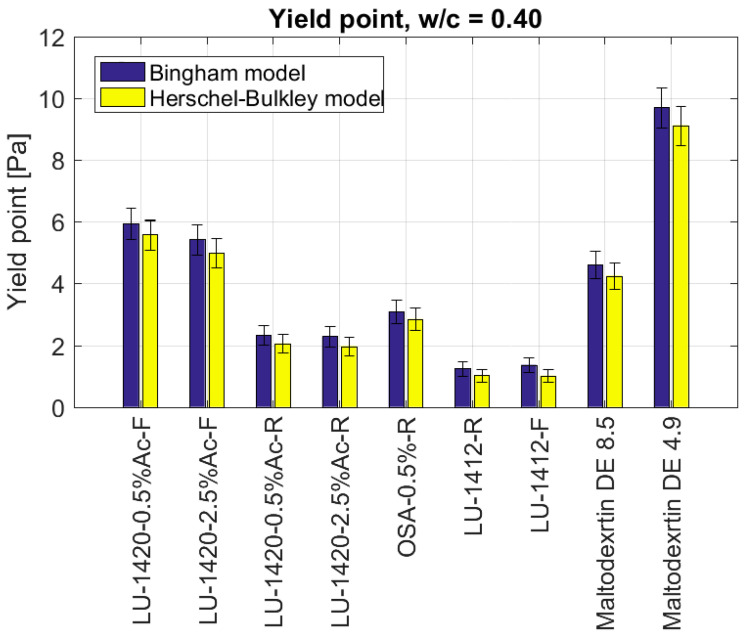
Yield points of cement slurries depending on the type of admixture in the form of modified starch for w/c = 0.40.

**Figure 5:**
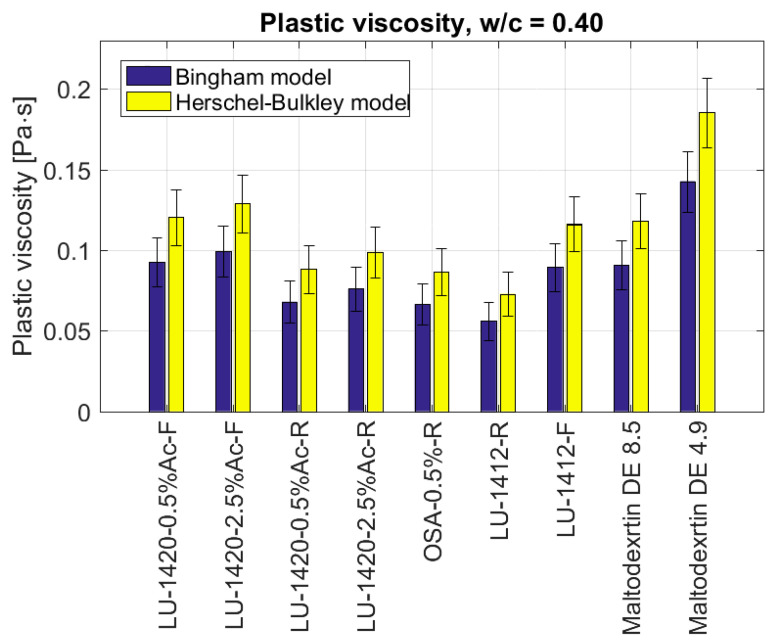
Plastic viscosities of cement slurries depending on the type of admixture in the form of modified starch for w/c = 0.40.

Figure 4 and Figure 5 present the results of the yield point and plastic viscosity for nine admixtures of starch hydrolysates for w/c = 0.4.

The lowest values of the yield point (Figure 4) were recorded for the admixtures of the retentate LU-1412-R and the filtrate LU-1412-F ($\tau_{0}=1.0\;\mathrm{Pa}$), the retentate LU-1420-2.5%Ac-R ($\tau_{0}=2.0\;\mathrm{Pa}$), the retentate LU-1420-0.5%Ac-R ($\tau_{0}=2.0\;\mathrm{Pa}$) and the retentate OSA-0.5%-R ($\tau_{0}=2.6\;\mathrm{Pa}$). Higher values of the yield point were obtained for the Maltodextrin DE 8.5 ($\tau_{0}=4.3\;\mathrm{Pa}$), the filtrate LU-1420-2.5%Ac-F ($\tau_{0}=5.0\;\mathrm{Pa}$) and the filtrate LU-1420-0.5%Ac-F ($\tau_{0}=5.6\;\mathrm{Pa}$). The highest value of the yield point ($\tau_{0}=9.1\;\mathrm{Pa}$) was noted for the addition of the Maltodextrin DE 4.9.

The lowest values of plastic viscosity (Figure 5) were observed for the retentate LU-1412-R ($\eta_{pl}=0.073\;\mathrm{Pa}\cdot s$), the retentate OSA-0.5%-R $\left(\eta_{pl}=0.087\;\mathrm{Pa}\cdot s\right)$, the retentate LU-1420-0.5%Ac-R ($\eta_{pl}=0.088\;\mathrm{Pa}\cdot s$) and the LU-1420-2.5%Ac-R ($\eta_{pl}=0.099\;\mathrm{Pa}\cdot s$). The admixtures of the filtrates LU-1420-0.5%Ac-F and LU-1420-2.5%Ac-F and of the filtrate LU-1412-F and Maltodextrin DE 8.5 gave similar results ($\eta_{pl}=0.12\;\mathrm{Pa}\cdot s$). The highest value of plastic viscosity was achieved with the Maltodextrin DE 4.9 ($\eta_{pl}=0.19\;\mathrm{Pa}\cdot s$).

### 3.3. Compressive Strength of Cement Mortars Admixed with Modified Starches

**Figure 6:**
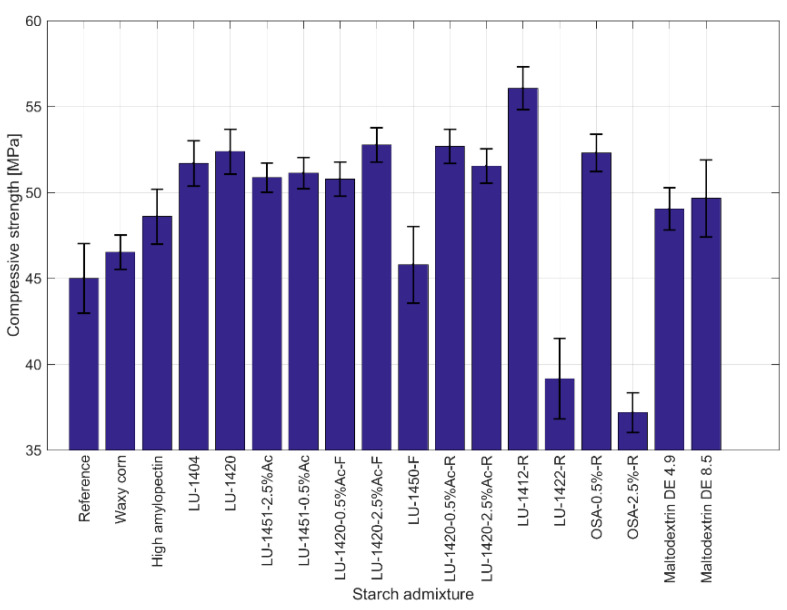
Compressive strengths of cement-mortar beams, depending on the type of admixture used.

The strength tests assumed the use of cement mortar samples made with admixtures of 17 different starch derivatives. The compressive strengths of the samples were tested 28 days after producing the beams. The test results, along with standard deviations, are presented graphically in Figure 6.

As in the rheological tests, the starches were divided into modified starches and starch hydrolysates. The test results were compared with the average compressive strengths of the beams produced without modifiers, i.e., the reference sample, which was 45 MPa.

For all the modified starch admixtures that were not starch hydrolysates, an increase in the 28-day compressive strength was noted. The highest increase occurred for the acetylated starch LU-1420 (compressive strength 52.4 MPa, increase of 16%), the oxidized starch LU-1404 (compressive strength 51.7 MPa, increase of 15%) and the gelling starch LU-1450-0.5%Ac (compressive strength 51.1 MPa, increase of 14%). A slightly smaller increase in the compressive strength occurred with the Waxy maize starch (compressive strength 46.5 MPa, increase of 3%) and the High amylopectin starch (compressive strength 48.6 MPa, increase of 8%).

The use of admixtures made of starch hydrolysates resulted in both decreases and increases in the compressive strengths of the cement mortar beams, depending on the derivative used.

Strength gains occurred for the admixtures of the retentate LU-1412-R (compressive strength 56.1 MPa, increase of 25%), filtrate LU-1420-2.5%Ac-F (compressive strength 52.8 MPa, increase of 17%), retentate LU-1420-0.5%Ac-R (compressive strength 52.7 MPa, increase of 17%), retentate OSA-0.5%-R (compressive strength 52.3 MPa, increase of 16%) and retentate LU-1420-2.5%Ac-R (compressive strength 51.5 MPa, increase of 14%).

Smaller increases in the compressive strength were found for the admixtures of the Maltodextrin DE 8.5 (compressive strength 49.7 MPa, increase of 10%) and Maltodextrin DE 4.9 (compressive strength 49.0 MPa, increase of 9%). A slight increase in the compressive strength for the admixture of the filtrate LU-1412-F (compressive strength 45.8 MPa, increase of 2%) was found.

Declines in compressive strength were noticed for the retentate LU-1422-R (compressive strength 39.2 MPa, decrease of 13%) and the retentate OSA-2.5%-R (compressive strength 37.2 MPa, decrease of 17%).

## 4. Discussion

All the tested starch hydrolysates caused a significant decrease in both the yield point and the plastic viscosity of the samples of cement slurry. The lowest values of the yield point were obtained for the fractions of the retentates. Among all the hydrolysates, the smallest reduction in the yield point was observed for the maltodextrins, particularly Maltodextrin DE = 4.9.

These effects may have been due to the fact that the fractions of retentates contained the remains of the enzyme used in the process of enzymatic hydrolysis; thus, their surface activity was higher than that of the respective filtrate fractions [28]. This directly affected the water content present in cement grains.

Considering the plastic viscosity, most of the starch hydrolysates reduced it significantly and its lowest values were also obtained for the fractions of the retentates.

It is worth noting that even when the w/c was reduced to 0.40, none of the starch hydrolysates reached the yield point for the reference slurry, determined by the value of the water–cement ratio equal to 0.50. For the retentates, the reduction in the yield point was above 80%.

During the rheological tests, it was noted that the remaining modified starches did not change the yield points of the cement slurries or, in most cases, increase it. The same conclusions can also be drawn by analysing the obtained values of plastic viscosity. Due to the compaction of the mix, it was impossible to lower the w/c ratio to 0.40.

The analysis of the above data shows that depending on the method of starch modification, different effects on the behaviour of cement mixes were obtained. Hydrolysed starches cause significant fluidisation. The best results were obtained for the fractions of the retentates. Furthermore, the number of acetyl groups of starch is important for fluidisation. However, it is not clear whether starches with more or fewer groups provide greater fluidisation, as this depends on the type of starch. The molecular weight of the starch seems to be a critical point affecting fluidisation [20]. The tested modified starches, which were not hydrolysates, did not significantly affect the rheological parameters (Figure 1 and Figure 2) of the cement mortars, because higher-molecular-weight polymers have the ability to agglomerate, thereby causing the cement mix to thicken, while lower molecular weight polymers (such as starch hydrolysates) tend to adsorb on the surface of the cement grains, causing a dispersing effect, thereby reducing the plastic viscosity of the mix. The influence of the polysaccharide structure on cement hydration kinetics has been described in the literature.

Differences in the degree of the fluidisation of cement mixes obtained due to modification with starch preparations or starch hydrolysates result directly from their chemical structure—the degree of polymerisation of admixtures. Starch hydrolysates have shorter polymer chains; hence, their saccharification degree is higher, which, consequently, affects the fluidisation of cement mixes. Furthermore, not without significance is the fact that the surface activity of starch hydrolysates, especially retentates, is higher compared to their initial preparations [29,35].

Starch hydrolysates change the compressive strengths of cement-mortar beams. However, they do not lead to an increase in compressive strength in all cases. For preparations with OSA 2.5% and retentate thickener AD, a decrease in the compressive strengths of the samples was noticed. These substances were correlated with the occurrence of cement-setting problems; the samples were disassembled after 2 days, which may have reduced their compressive strength. The remaining samples were disassembled after 24 h. Tan H. et al. [36] also noticed a decrease in the strengths of concretes doped with corn starch and carboxymethyl starch. In the cases of other modified starches, increases in the compressive strengths of the samples was observed in all cases. Other authors also observed increases in the strengths of cement composites as a result of doping them with starches. For example, Akindahunsi and Uzoegbo [37] showed the positive effects of corn starch and cassava on the strengths of cement concretes (increase of 5%). The authors also showed that compression influences starch polymerization; the higher it is, the greater the compressive strength. However, the author’s own research [38] showed that the spaces between the grains of starch hydrolysates and cement slurry are smaller, which may be associated with stronger bonds between the molecules. Moreover, it was noted that sugars exhibit the ability to bind to the cement phase, depending on the presence of the carboxyl group (i.e., oxidated starch) (Figure 6).

## 5. Conclusions

- It was found that the consistency and strength of cement mortars are affected by both the type of modification and the length of the chemical chain of starch.
  The highest strength gains occurred for the admixtures of retentate LU-1412-R (increase of 25%). Declines in compressive strength were noted in the retentate LU-1422-R (decrease by 13%) and in the retentate OSA-2.5%-R (decrease of 17%).
- It was demonstrated that all the tested starch hydrolysates caused a significant decrease in the yield points and plastic viscosities of the cement slurries and an increase in the flow diameters of the cement mortars and concretes. The highest degree of fluidisation, which was also confirmed by the viscometer tests, and the lowest values of the yield point and plastic viscosity were obtained for the fractions of the retentates. The thickening of the cement mix and an increase in the yield point were observed for the potato starches that were not starch hydrolysates.
  The differences in the degrees of fluidisation of the cement mixes obtained due to the modifications with starch preparations or starch hydrolysates resulted directly from their chemical structure—the degree of polymerisation of the admixtures. Starch hydrolysates have shorter polymer chains; hence, their saccharification degree is higher, which, consequently, affects the fluidisation of cement mixes.
- Taking into account both the compressive strength and the rheological parameters, the best results were obtained with the LU-1412R.

Further investigations may be required to determine the effects of both the type and the amount of the added admixture on the course of the bonding process of cement composites.

## Figures and Tables

**Table 1:** Composition of test used for viscosity measurement.

w/c	Cement (g)	Water (g)	Starch (g)
0.5	50	25	0.25
0.4	50	20	

## Data Availability

The data that support the findings of this study are available from the corresponding author upon reasonable request.
